# Deficient mismatch repair system in patients with sporadic advanced colorectal cancer

**DOI:** 10.1038/sj.bjc.6604867

**Published:** 2009-01-22

**Authors:** M Koopman, G A M Kortman, L Mekenkamp, M J L Ligtenberg, N Hoogerbrugge, N F Antonini, C J A Punt, J H J M van Krieken

**Affiliations:** 1 Department of Medical Oncology, Radboud University Nijmegen Medical Centre, Nijmegen, The Netherlands; 2 Department of Pathology, Radboud University Nijmegen Medical Centre, Nijmegen, The Netherlands; 3 Department of Human Genetics, Radboud University Nijmegen Medical Centre, Nijmegen, The Netherlands; 4 Department of Biometrics, Netherlands Cancer Institute, Amsterdam, The Netherlands

**Keywords:** mismatch repair, advanced colorectal cancer, chemotherapy, incidence, prognosis

## Abstract

A deficient mismatch repair system (dMMR) is present in 10–20% of patients with sporadic colorectal cancer (CRC) and is associated with a favourable prognosis in early stage disease. Data on patients with advanced disease are scarce. Our aim was to investigate the incidence and outcome of sporadic dMMR in advanced CRC. Data were collected from a phase III study in 820 advanced CRC patients. Expression of mismatch repair proteins was examined by immunohistochemistry. In addition microsatellite instability analysis was performed and the methylation status of the *MLH1* promoter was assessed. We then correlated MMR status to clinical outcome. Deficient mismatch repair was found in only 18 (3.5%) out of 515 evaluable patients, of which 13 were caused by hypermethylation of the *MLH1* promoter. The median overall survival in proficient MMR (pMMR), dMMR caused by hypermethylation of the *MLH1* promoter and total dMMR was 17.9 months (95% confidence interval 16.2–18.8), 7.4 months (95% CI 3.7–16.9) and 10.2 months (95% CI 5.9–19.8), respectively. The disease control rate in pMMR and dMMR patients was 83% (95% CI 79–86%) and 56% (30–80%), respectively. We conclude that dMMR is rare in patients with sporadic advanced CRC. This supports the hypothesis that dMMR tumours have a reduced metastatic potential, as is observed in dMMR patients with early stage disease. The low incidence of dMMR does not allow drawing meaningful conclusions about the outcome of treatment in these patients.

In recent years the median overall survival (OS) for patients with advanced colorectal cancer (CRC) has significantly improved, mainly because of the availability of cytotoxic agents such as oxaliplatin and irinotecan, and the targeted drug bevacizumab (Punt, 2004). Despite these improvements cure is only rarely achieved, and not all patients respond to chemotherapy. Therefore, there is a need for predictive and prognostic tests that identify patients who may or may not benefit from systemic treatment.

Deficient mismatch repair (dMMR) is one of the genetic pathways that is involved in the development of CRC (Aaltonen *et al*, 1993; Ionov *et al*, 1993). Microsatellites are repetitive units in DNA. In normal circumstances insertions or deletions in these regions are repaired by the MMR system, which consists of several cooperating MMR proteins. Dysfunction of this system is causing dMMR. Known MMR gene products are: MLH1, MSH2, MSH6 and PMS2. A germ line mutation in one of these corresponding genes, most often MLH1 or MSH2, is the cause of dMMR in patients with Lynch syndrome, formerly Hereditary Non Polyposis Colorectal Cancer (HNPCC), which comprises 0.8–5% of all CRCs (Mecklin, 1987; Rodriguez-Bigas *et al*, 1997; Cunningham *et al*, 2001). Deficient mismatch repair is also observed in 10–20% of patients with sporadic CRC, usually caused by *MLH1* promoter hypermethylation (Lothe *et al*, 1993; Kane *et al*, 1997; Cunningham *et al*, 1998; Peltomaki, 2003). Microsatellite analysis is the gold standard for the detection of dMMR in patients with a suspicion of Lynch syndrome, as well as in tumours with indecisive results of IHC. Lindor *et al* (2002) showed that immunohistochemistry (IHC) in colorectal tumours for MLH1 and MSH2 provides a rapid, cost-effective, sensitive (92.3%) and highly specific (100%) method for screening for DNA MMR defects, which was recently confirmed by our group (Overbeek *et al*, 2008).

Colorectal carcinomas with dMMR show several distinct pathological features, such as a location in the proximal colon, a poor histological differentiation, high numbers of tumour infiltrating lymphocytes, and are more often of mucinous type. Moreover patients with dMMR tumours have a better prognosis compared to patients with proficient MMR (pMMR) tumours (Sankila *et al*, 1996; Gryfe *et al*, 2000; Samowitz *et al*, 2001; Ward *et al*, 2001; Popat *et al*, 2005). Several studies investigated if chemosensitivity is implied in the better prognosis of patients with a dMMR tumour. *In vitro* studies have shown dMMR cell lines to be resistant to 5-fluorouracil (5FU) (Carethers *et al*, 1999; Arnold *et al*, 2003), but not to oxaliplatin (Fink *et al*, 1996; Sergent *et al*, 2002) or irinotecan (Magrini *et al*, 2002). In patients receiving adjuvant chemotherapy for CRC, conflicting results have been reported on the correlation between dMMR and outcome in retrospective studies (Elsaleh and Iacopetta, 2001; Ribic *et al*, 2003; Carethers *et al*, 2004; Benatti *et al*, 2005). Therefore, the ASCO 2006 and European 2007 guidelines do not recommend the use of dMMR as a prognostic and/or predictive marker in this setting (Locker *et al*, 2006; Duffy *et al*, 2007). Only few data from small, non-randomised studies are available on the function of dMMR in patients with advanced CRC (Popat *et al*, 2005). In most of these studies sporadic and hereditary CRC were not clearly differentiated. Histopathological and clinical differences between tumours with a mutation of a MMR gene and tumours with hypermethylation of the *MLH1* promoter as a cause of dMMR have been described (Benatti *et al*, 2005; Jass, 2007), and therefore it seems reasonable to distinguish between these two types of dMMR tumours regarding response to therapy and survival (Poynter *et al*, 2008).

This is the first randomised study with chemotherapy in patients with advanced CRC which evaluates the incidence of dMMR and its correlation with clinical outcome.

## Materials and methods

### Study population

Tumour tissue was obtained from patients enrolled in a randomised phase III study, the CAIRO study of the Dutch Colorectal Cancer Group (DCCG), registered with ClinicalTrials.gov with the number NCT00312000, of which the results on survival have been published (Koopman *et al*, 2007). In this study 820 patients were randomised between first-line capecitabine, second-line irinotecan and third-line capecitabine+oxaliplatin (sequential treatment arm) *vs* first-line capecitabine+irinotecan, and second-line capecitabine+oxaliplatin (combination treatment arm). The primary endpoint was OS, and secondary endpoints included response rate, and progression-free survival (PFS). Assessment of tumour response was scheduled every three cycles (9 weeks) according to RECIST criteria (Therasse *et al*, 2000). Follow-up after completion of treatment was carried out every 3 months until death. For this study formalin-fixed paraffin-embedded material of the primary tumour and normal tissue was obtained from 515 patients out of 803 eligible patients of the CAIRO study. No tissue material was obtained in 288 patients because a resection of the primary tumour was not performed, or there was insufficient or non-available material.

### Histology and pathology

Histopathologic evaluation was carried out by two independent observers, and in case of discordant results, the opinion of a third observer (pathologist: JvK) was final. Histopathological subtype and grade of differentiation were determined according to WHO criteria (Jass *et al*, 1990). Tumours were classified as right-sided (proximal of the splenic flexure), left-sided (distal of the splenic flexure) or rectal.

### Immunohistochemistry

IHC was performed on formalin-fixed paraffin-embedded tissue. Of each paraffin-embedded block a 2 mm punch for assembling tissue microarrays (TMA's) was accomplished as previously described (Hendriks *et al*, 2003). Four 4 μm slides were assessed of every TMA and mounted on glass. The TMA slides were deparaffined and afterwards the tissues were rehydrated using xylene and ethanol. Water and phosphate-buffered saline (PBS) were used for washing of the slides. Endogenous peroxidase activity was blocked with 3% hydrogen peroxide in PBS for 30 min and slides were washed with water, after which heat-induced epitope retrieval was performed. The slides were stained with antibodies against MLH1 (clone G168-15; BD Biosciences, San Jose, CA, USA), MSH2 (clone GB12; Calbiochem, Darmstadt, Germany), MSH6 (clone 44; BD Biosciences) and PMS2 (clone A16-4; BD Biosciences). The scoring was performed by two blinded observers, and if the slide scoring was not unambiguous, the opinion of a third observer (pathologist: JvK) was final. Staining pattern of the MMR proteins was evaluated by using the normal epithelial, stromal and inflammatory cells as internal control. Protein expression was scored positive if at least one cancer cell nucleus showed staining, negative if none of the tumour cells showed staining with positive internal control, and not applicable if neither tumour nor stromal cells showed protein expression. In case of absence of MMR protein or not interpretable results of IHC, the IHC stainings on TMA were repeated on whole tissue slides for final scoring.

### Microsatellite instability analysis

Microsatellite instability (MSI) analysis was performed for tumours of which the final IHC staining was not interpretable or with a negative staining for at least one of four mismatch repair proteins. In addition a random sample of 54 tumours was taken from the pMMR tumours. DNA was extracted from formalin-fixed paraffin-embedded tissues of tumour mucosa and corresponding normal mucosa by a standard procedure. Areas containing >50% tumour cells were selected by microscopic evaluation on a reference slide stained with H&E. Slides (50 μm thick) were made, and if necessary tumour cells were prepared using a scalpel. MSI status was determined by PCR and GeneScan analysis using two microsatellite markers (BAT 25 and BAT 26). If only one of these markers showed instability, the analysis was extended with four additional markers (BAT 40, D2S123, D5S346, D17S250) (Boland *et al*, 1998; Samowitz *et al*, 1999). A tumour was defined dMMR if at least two of the six markers showed instability, or pMMR if none of the markers showed any shift in mobility. MSI-low tumours with only one of the markers showing instability were included in the pMMR category. For the distinction between dMMR tumours caused by hypermethylation of the *MLH1* promoter and mutation of one of the mismatch repair genes, dMMR tumours were further analysed for hypermethylation of the *MLH1* promoter (Bettstetter *et al*, 2007).

### Hypermethylation of *MLH1* promoter

The DNA methylation status of the *MLH1* promoter region was determined after bisulphite treatment of the DNA using the EZ DNA methylation KIT, ZYMO Research (Orange, CA, USA), as described before (Overbeek *et al*, 2007).

### Statistical analysis

Patients were divided into three groups: pMMR tumours, dMMR tumours caused by hypermethylation of the *MLH1* promoter and dMMR tumours without hypermethylation of the *MLH1* promoter. Survival analysis was performed for patients with pMMR tumours *vs* dMMR tumours caused by hypermethylation of the *MLH1* promoter and *vs* the total group of patients with a dMMR tumour, respectively. The association between dMMR and patient or tumour characteristics was investigated with an univariate logistic regression model. Patients were considered evaluable for response if they had completed at least three cycles of chemotherapy. Disease control was defined by stable disease with a duration of ⩾4 months or partial response or complete response. Differences in response and disease control rates were analysed by a *χ*^2^ (univariate) model. The PFS was calculated for first-line treatment, from the date of randomization to the first observation of disease progression or death from any cause reported after first-line treatment. OS and PFS curves were estimated using the Kaplan–Meier method and compared using a Cox proportional hazard model. All tests were two-sided and *P*-values of less than 0.05 were considered significant. All data received before May 2008 are included in this report with a median follow-up of 43 months. All analyses were performed using SAS 9.1 and S-plus 6.2 software.

## Results

### IHC, MMR and hypermethylation of the *MLH1* promoter

**Figure 1:**
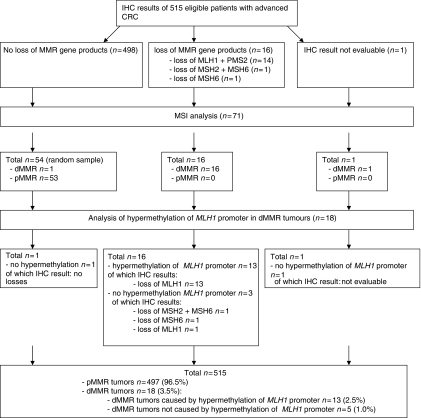
IHC results, MSI analysis and hypermethylation *MLH1* promoter.

Figure 1 shows the results on IHC, MMR and hypermethylation of the *MLH1* promoter. Samples of 515 eligible patients were available for IHC. In 498 tumours no loss for MMR gene products was observed, 14 tumours showed loss of MLH1 in combination with PMS2, 2 tumours showed loss of MSH6 of which one in combination with MSH2, and in one tumour the IHC staining was not evaluable. All these 17 tumours with loss/not evaluable IHC result of at least one MMR protein turned out to be dMMR by MSI analysis. In 54 tumours without loss of MMR gene products (random sample), dMMR was detected in 1 tumour by MSI analysis, resulting in a total of 18 dMMR tumours (3.5%). Hypermethylation of the *MLH1* promoter was found in 13 out of these 18 tumours, all with protein loss of MLH1 by IHC. This resulted in 515 patients for the analysis: 18 with a dMMR tumour (3.5%), of which 13 patients with a dMMR tumour caused by hypermethylation of the *MLH1* promoter, and 497 patients with a pMMR tumour.

### Patient characteristics and MMR status

Patient characteristics of the three groups of patients are presented in Table 1. The median age of the included patients was 63 years (range 31–81). Significant differences between the group of patients with dMMR caused by hypermethylation of the *MLH1* promoter and the pMMR group were seen for the location (*P*<0.0001) and differentiation grade of the primary tumour (*P*=0.025). Patients with a dMMR tumour not caused by *MLH1* promoter hypermethylation were younger than patients with a dMMR tumour caused by *MLH1* promoter hypermethylation (*P*=0.0051). The univariate analysis showed an association of poorly differentiated features with an increased probability of exhibiting dMMR, patients with a poor/undifferentiated tumour have a 3.9 times increased risk of exhibiting dMMR compared to well/moderate differentiated tumours (*P*=0.025).

### OS and PFS in relation to MMR status of the tumour

**Figure 2:**
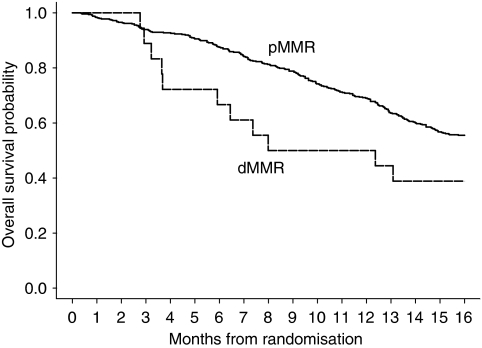
OS by MMR status.

At the time of this analysis 447 patients have died, of which 15 patients exhibited dMMR. Table 2 presents the median OS and PFS for the pMMR and dMMR group of patients. The median OS was 17.9 months (95% CI 16.2–18.9), 7.4 months (95% CI 3.7–16.9) and 10.2 months (95% CI 5.9–19.8) for patients with a pMMR tumour, a dMMR tumour caused by hypermethylation of the *MLH1* promoter, and the total group of patients with a dMMR tumour, respectively (Table 2; Figure 2). These differences were statistically not significant (Table 2). In the sequential treatment arm, the median OS for these groups of patients was 17.2 months (95% CI 14.7–18.8), 12.4 months (95% CI 3.2–->) and 12.7 months ((95% CI 7.4–22.2), respectively, and in the combination treatment arm 18.3 months (95% CI 16.2–20.6), 6.2 months (95% CI 3.6–31.3) and 6.2 months (95% CI 3.6–31.3), respectively. All these differences were statistically not significant (Table 2).

**Figure 3:**
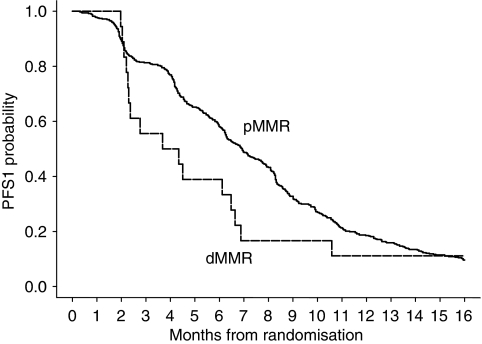
PFS by MMR status.

The median PFS for patients with a pMMR tumour, dMMR tumours caused by hypermethylation of the *MLH1* promoter, and the total group of patients with a dMMR tumour, was 6.9 months (95% CI 6.3–7.7), 4.3 months (95% CI 2.4–6.6) and 4.0 months (95% CI 2.3–6.5), respectively (Figure 3). In the sequential treatment arm the median PFS for these groups of patients was 5.8 months (95% CI 4.9–6.3), 6.6 months (95% CI 2.2–->) and 4.2 months (95% CI 2.2–10.6), respectively, and for the combination treatment arm 8.3 months (95% CI 7.6–8.7), 4.0 months (95% CI 2.3–6.5) and 8.2 months (95% CI 7.4–8.5), respectively. The pair-wise comparison with pMMR and the two groups of dMMR patients was not significant in the first comparison (*P*=0.06), and significant in the second comparison (*P*=0.02).

### Tumour response in relation to MMR status

A total of 511 patients received first-line therapy. Deficient mismatch repair was observed in 18 patients, of whom 13 patients showed hypermethylation of the *MLH1* promoter. Of these 511 patients, dMMR was detected in 16 out of 469 patients evaluable for response on first-line therapy, in 8 out of 288 patients evaluable for response on second-line therapy and in none of 96 patients evaluable for response on third-line therapy. The response and PFS analyses are only shown for first-line due to the low number of patients with dMMR tumours in second- and third-line treatment (Tables 2 and 3). The overall response rate in first-line treatment for pMMR, dMMR caused by hypermethylation of the *MLH1* promoter and the total group of patients with a dMMR tumour was 31, 33 and 25%, respectively. Results on disease control in first-line treatment in the pMMR group, the dMMR group caused by hypermethylation of the *MLH1* promoter and the total group of patients with a dMMR tumour, were 83, 58 and 56%, respectively (Table 3).

## Discussion

This is the first large randomised study in advanced CRC patients in which the incidence of dMMR and the correlation between sporadic dMMR and the outcome of chemotherapy is investigated. The incidence of dMMR was low (18 out of 515 patients, 3.5%). In 13 of these patients (2.5%) dMMR was caused by *MLH1* promoter hypermethylation.

The incidence is lower as the previously reported incidence of 10–20% dMMR in sporadic CRC's (Lothe *et al*, 1993; Peltomaki, 2003). We ruled out the possibility of an underestimation of dMMR due to technical failure for several reasons. Previously it has been shown that IHC is an accurate, highly specific and sensitive method for detecting dMMR (Ward *et al*, 2001; Lindor *et al*, 2002; Overbeek *et al*, 2008). We performed IHC staining on TMA's (Hendriks *et al*, 2003), and confirmed the results on whole tissue slides and by MSI analysis. Furthermore, MSI analysis in a random sample of patients with pMMR as determined by IHC did confirm the results.

The low incidence in our series may be explained by a reduced potential in stages I–III dMMR patients to develop metastases (Lim *et al*, 2004; Malesci *et al*, 2007), as most of the previously reported incidences were observed in these earlier stages of CRC (Popat *et al*, 2005). Thus, as a consequence the incidence of dMMR in advanced CRC studies will be lower than 10–20%. Data on the incidence in stage IV patients are scarce and are mostly derived from small, non-randomised studies, with a reported incidence between 0.5 and 21% (Kochhar *et al*, 1997; Samowitz *et al*, 2001; Liang *et al*, 2002; Ricciardiello *et al*, 2005; Malesci *et al*, 2007; Muller *et al*, 2008). A low dMMR incidence of 0 and 2.7% was found in advanced CRC patients with resected liver (Haddad *et al*, 2004) and lung metastases (Melloni *et al*, 2006), respectively. An incidence of 4.4% of MLH1/MSH2 loss was reported in a large cohort of advanced CRC patients (Braun *et al*, 2008).

Our results on the correlation of dMMR with clinical outcome should be interpreted with caution due to the low number of dMMR patients. We found a non-significant decrease in median OS of 17.9 *vs* 10.2 months, and in median PFS of 6.9 *vs* 4.0 months in pMMR *vs* dMMR patients treated with chemotherapy, with a decreased disease control rate of 83 *vs* 56%, respectively. These results are difficult to compare with previously reported results for several reasons. First, dMMR has been tested as a predictive marker in advanced CRC in only one randomised study (Muller *et al*, 2008). In this study with 474 patients, tumour tissue was available from 104 patients of whom only 4 patients treated with 5FU and oxaliplatin tested positive for dMMR. Second, we used capecitabine as a fluoropyrimidine. It is yet unknown whether the outcome of 5FU and capecitabine differs in respect to the MMR status of patients. Third, in contrast to most studies we differentiated between dMMR caused by hypermethylation of the *MLH1* promoter, dMMR without *MLH1* promoter hypermethylation, and pMMR. Hereditary and sporadic dMMR tumours may differ in terms of pathologic features, underlying molecular alterations, and prognosis, and it is known that patients with dMMR tumours with overlapping hypermethylator phenotype have a worse clinical outcome compared to dMMR tumours without promoter hypermethylation (Hawkins *et al*, 2002; Ward *et al*, 2003; McGivern *et al*, 2004; Benatti *et al*, 2005; Johnson *et al*, 2005; Jass, 2007).

A reduced chemosensitivity was observed in CRC cell lines with *MLH1* promoter hypermethylation treated with fluoropyrimidines (Arnold *et al*, 2003). Although we found a decreased disease control rate in dMMR patients, our numbers are too small to draw meaningful conclusions. Definite proof for a reduced chemosensitivity of advanced dMMR tumours can only be obtained from prospective randomised studies with chemotherapy *vs* observation. However studies with this design are considered unethical given the benefit of chemotherapy. We considered a control group within the same prospective trial which therefore was selected, treated and monitored in exactly the same way as the most appropriate alternative.

In conclusion, dMMR caused by hypermethylation of the *MLH1 –*promoter is a rare event in advanced CRC patients. This supports the hypothesis that dMMR tumours have a reduced metastatic potential. Given the low incidence of dMMR in advanced CRC patients, our results do not allow any meaningful conclusions on the correlation between dMMR status and clinical outcome. This topic should be addressed by a pooled analysis of multiple trials. The low incidence of dMMR does not justify the need for standard dMMR testing in advanced CRC patients.

## Figures and Tables

**Table 1:** Patient characteristics according to MMR status

**Number of eligible patients**	**Group 1 pMMR (*n*=497)**	**Group 2 dMMR *MLH1* hypermethylation (*n*=13)**	**Group 3 dMMR no *MLH1* hypermethylation (*n*=5)**	***P*-value^#^ (1 *vs* 2)**	***P*-value^#^ (2 *vs* 3)**
*Age*					
⩽50 years	51 (10%)	0 (0%)	2 (40%)		
Median (range)	63 (31–81)	70 (54–78)	57 (35–64)	0.053	0.0051
					
*Gender*					
Male	315 (63%)	7 (54%)	4 (80%)	0.49	0.29
					
*Location of the primary tumour*					
Colon—left	164 (33%)	—	—	<0.0001	—
Colon—right	122 (25%)	13 (100%)	5 (100%)		
Rectosigmoid	30 (6%)	—	—		
Rectum	161 (32%)	—	—		
Unknown	20 (4%)	—	—		
					
*Histology of primary tumour*					
Adenocarcinoma	438 (88%)	10 (77%)	2 (40%)	0.10	0.14
Mucinous adenocarcinoma	38 (8%)	3 (23%)	3 (60%)		
Adenosquamous carcinoma	4 (<1%)	—	—		
Undifferentiated carcinoma	3 (<1%)	—	—		
Unknown	14 (3%)	—	—		
					
*Differentiation grade*					
Well/moderate	259 (52%)	3 (23%)	2 (40%)	0.025	0.48
Poor/undifferentiated	222 (45%)	10 (77%)	3 (60%)		
Unknown	16 (3%)	—	—		
					
*Diagnosis of metastases*					
<12 months before randomization	292 (59%)	8 (62%)	1 (20%)	0.84	0.11
< 6 months before randomization	244 (49%)	7 (54%)	1 (20%)	0.77	0.18
					
*Number of sites involved*					
1 site of metastases	241 (48%)	7 (54%)	1 (20%)	0.73	0.18
>1 site of metastases	250 (50%)	6 (46%)	4 (80%)		
Unknown	6 (1%)	—	—		
					
Primary tumour involved at start chemotherapy	62 (12%)	3 (15%)	2 (40%)	0.37	0.54
Previous adjuvant therapy	82 (17%)	2 (15%)	2 (40%)	0.91	0.28
Resection of primary tumour	488 (98%)	13 (100%)	5 (100%)	0.49	—

^#^*P*-value logistic regression.

**Table 2:** OS and PFS according to MMR status with the number of events in italic

**Number of patients**	**PMMR (*n*=497)**	**dMMR *MLH1* hypermethylation (*n*=13)**	**dMMR total (*n*=18)**	***P*-value^#^** **pMMR *vs* dMMR hypermethylation**	***P*-value^#^** **pMMR *vs* dMMR total**
*Overall survival (months)*					
Median (95% CI)	17.9 (16.2–18.9)	7.4 (3.7–16.9)	10.2 (5.9–19.8)	0.27	0.41
	*n=440*	*n=11*	*n=15*		
Sequential treatment	17.2 (14.7–18.8)	12.4 (3.2––>)	12.7 (7.4–22.2)	0.58	0.47
Median (95% CI)	*n=230*	*n=4*	*n=7*		
Combination treatment	18.3 (16.2–20.6)	6.2 (3.6–31.3)	6.2 (3.6–31.3)	0.25	0.58
Median (95% CI)	*n=210*	*n=7*	*n=8*		
					
*PFS (months)*					
Median (95% CI)	6.9 (6.3–7.7)	4.3 (2.4–6.6)	4.0 (2.3–6.5)	0.85	0.28
	*n=490*	*n=12*	*n=17*		
Sequential treatment	5.8 (4.9–6.3)	6.6 (2.2–->)	4.2 (2.2–10.6)	0.27	0.72
Median (95% CI)	*n=247*	*n=4*	*n=7*		
Combination treatment	8.3 (7.6–8.7)	4.0 (2.3–6.5)	4.0 (2.3–6.5)	0.06	0.02
Median (95% CI)	*n=243*	*n=8*	*n=10*		

^#^*P*-value Cox regression.

**Table 3:** Best overall response in first-line treatment according to MMR status

**Patients evaluable for response**	**pMMR** **(*n*=453)**	**dMMR MLH1-hypermethylation** **(*n*=12)**	**dMMR total** **(*n*=16)**	***P*-value^#^** **pMMR *vs* dMMR hypermethylation**	***P*-value^#^** **pMMR *vs* dMMR total**
*Best overall response*					
Complete response	16 (4%)	—	—		
Partial response	123 (27%)	4 (33%)	4 (25%)		
Stable disease	235 (52%)	3 (25%)	5 (31%)		
Progressive disease	79 (17%)	5 (42%)	7 (44%)		
					
Response rate (95% CI)	31% (27–35%)	33% (10–65%)	25% (7–52%)	0.84	0.63
	*n*=139	*n*=4	*n*=4		
Disease control (95% CI)	83% (79–86%)	58% (28–85%)	56% (30–80%)	0.031	0.008
	*n*=374	*n*=7	*n*=9		
					
*Sequential treatment*	*n*=239	*n*=5	*n*=8		
					
Response rate (95% CI)	17% (13–23%)	40% (5–85%)	25% (3–65%)	0.19	0.57
	*n*=41	*n*=2	*n*=2		
Disease control (95% CI)	76% (70–81%)	60% (15–95%)	50% (16–84%)	0.40	0.09
	*n*=182	*n*=3	*n*=4		
					
*Combination treatment*	*n*=214	*n*=7	*n*=8		
					
Response rate (95% CI)	46% (39–53%)	29% (4–71%)	25% (3–65%)	0.46	0.30
	*n*=98	*n*=1	*n*=2		
Disease control (95% CI)	90% (85–93%)	57% (18–90%)	63% (25–92%)	0.033	0.048
	*n*=193	*n*=4	*n*=5		

^#^*P*-value *χ*^2^.
